# A comparative analysis of cycle threshold (Ct) values from Cobas4800 and AmpFire HPV assay for triage of women with positive hrHPV results

**DOI:** 10.1186/s12879-023-08737-4

**Published:** 2023-11-10

**Authors:** Yi Zhang, Wei Zhang, Hui Du, Xinfeng Qu, Yun Chen, Jianliu Wang, Ruifang Wu

**Affiliations:** 1 Department of Obstetrics and Gynecology, Peking University Shenzhen Hospital, Shenzhen Peking University- The Hong Kong University of Science and Technology Medical Center, Shenzhen, 518036 P. R. China; 2 Shenzhen Key Laboratory On Technology for Early Diagnosis of Major Gynecologic Diseases, Shenzhen, 518036 P. R. China; 3 Peking University Shenzhen Hospital, Shenzhen, China; 4 Department of Obstetrics and Gynecology, Peking University People’s Hospital, Beijing, China

**Keywords:** Human papillomavirus, Cobas4800, AmpFire, Circulating threshold, Viral load

## Abstract

**Background:**

To compare the triage performance of HPV viral loads reflected by cycle threshold values (CtV) from two different HPV testing assays: the PCR based Cobas4800 and the isothermal amplification based AmpFire assay.

**Methods:**

We used the data from a sub-study of The Chinese Multi-Center Screening Trial and analyzed the data of the cases positive in both Cobas4800 and AmpFire assays with recorded CtV. Spearman’s correlation was applied to analyze the association between CtV from AmpFire and Cobas4800 assays, as well as the correlation between CtV and the histological lesion grades. The 50th percentile of CtV was used as the cutoff to construct triage algorithms for HPV-positive cases. McNemar’s test was used to analyze the differences in sensitivity and specificity for detecting CIN2 + and CIN3 + in different triage algorithms.

**Results:**

Four hundred forty-six HPV positive women who had consistent HPV results from Cobas4800 and AmpFire in terms of the HPV genotype and reported Ct values were included in the analysis. The mean CtV of hrHPV tested by Cobas4800 and AmpFire were linear correlated. Direct association were showed between the severity of cervical lesions and the HPV viral loads reflected by CtV of hrHPV, HPV16, non-16/18 hrHPV and A9 group from both assays. HPV16/18 genotyping combined with low-CtV for non-16/18 hrHPV, especially A9 group, were demonstrated to be satisfactory in the sensitivity and specificity for detecting CIN2 + or CIN3 + .

**Conclusion:**

Ct value represented a good triage marker in both PCR-based and isothermal amplification HPV detection.

**Supplementary Information:**

The online version contains supplementary material available at 10.1186/s12879-023-08737-4.

## Background

Cervical cancer is a significant global health challenge. As the fourth most common cancer among women, it created 604,000 new cases of cervical cancer and 342,000 new deaths worldwide in 2020 [1]. The primary etiological factor underlying development of almost all cervical cancers is high-risk human papillomaviruses (hrHPV). However, only a small set of individuals with persistent hrHPV infection will ultimately develop high-grade cervical intraepithelial neoplasia that can potentially progress to cervical cancer if left untreated [2]. Effective prevention of cervical cancer need to vaccinate the young girls for hrHPV and treatment of the precancers detected from women through properly organized screening programs. Before HPV vaccination can cover all the world, effective screening is still the most realistic measure to control the current prevalence of cervical cancer, especially in the lower-and-middle-income-countries (LMIC).

HPV testing has been widely recognized as an effective primary screening method for cervical cancer, with high sensitivity and negative predictive value in detecting cervical lesions [3]. However, given the low probability of high-grade cervical lesions among HPV-positive women, it is not recommended to refer all hrHPV-positive individuals to colposcopy [4]. Therefore, an appropriate triage strategy is essential for identifying those individuals who require prompt colposcopy or treatment from those who need regular surveillance.

Cytology and HPV genotyping are the most common optional triage references used in cervical cancer screening programs [5]. Current guidelines recommend immediate referral of women who test positive for HPV16/18, with cytology triage for those positive for non-16/18 hrHPV [6]. However, cytology relies on cytologists with subjective skills and requires medical providers to collect cervical samples including exfoliated cells, which are usually not available in low-resource areas [5, 7]. In contrast, HPV genotypes are objective marks that can be obtained from HPV testing on self-collected samples and referable for triage in self-sampling-based cervical cancer screening programs in low-resource areas. However, genotyping may have diminishing returns in populations that have been immunized against certain HPV types, and the types included in a triage must be carefully balanced to avoid low specificity [5]. Recent data have shown that combining extended genotyping with cytology can provide a refined risk stratification for HPV-positive women [8].

HrHPV viral load is another molecular indicator that may tell the status of cervical cancer development [9–11]. Several commercial HPV assays can provide different indicators that reflect viral loads in the target samples as well as genotyping information. Studies have reported that setting more stringent HPV viral load cutoffs can help to improve the specificity of the assay to detect high-grade cervical lesions [12, 13].

The Cobas4800 assay is an FDA-approved real-time PCR-based HPV test that can provide both partial genotyping and cycle threshold values (CtV) for HPV-positive channels. The assay reports 14 high-risk HPV types in three channels: HPV16, HPV18, and a pooled 12 other hrHPV types (HPV-31, -33, -35, -39, -45, -51, -52, -56, -58, -59, -66, and -68). CtV from that assay represents the DNA amplification cycles required to make the fluorescence signal to reach a predetermined threshold. It is inversely correlated with the log amount of targeted DNA in the specimen [14, 15].

Our team has confirmed value of CtV from the Cobas4800 assay in positive triages by demonstrating that using HPV16/18 genotype and an appropriate cutoff of the Ct values for 12-hrHPV-in-pool as the triage protocol (the genotype/CtV protocol) could obtain a CIN2 + detection efficiency that was comparable to a protocol with combination of HPV16/18 genotypes and cytology (≥ ASCUSS) for non-16/18 hrHPV types [16–18]. The genotype/CtV protocol offered a pathway to full molecular screening and triage that is applicable to both self-collected and clinician-collected samples.

AmpFire is an isothermal, real-time fluorescent, and multiplex nucleic acid amplification method that allows detecting and genotyping 15 types of high-risk human papillomavirus (hrHPV) [19]. One of its advantages is that it detects HPV directly from the samples without needing the DNA extraction and purification procedures. Additionally, AmpFire can complete the testing procedures in about 2 h, which greatly reduces the time and const and makes it an ideal optional screening assay for “one-day” screening programs those are applied in remote or low-resource areas [19, 20]. Recent studies have shown that the AmpFire HPV assay has similar sensitivity and better specificity than the Cobas4800 assay in detecting ≥ grade 2 and ≥ grade 3 cervical intraepithelial neoplasia (CIN 2 + or CIN 3 +) on both self-collected and clinician-collected samples [20]. Similar to Cobas4800, AmpFire can also report the CtVs of the positive channels. However, since the AmpFire assay is isothermal, the CtV from AmpFire is simply a marker of reaction time (in minutes). To the best of our knowledge, no research has evaluated the role of AmpFire CtVs in triage of HPV-positive women.

In this study, we evaluated the performance of CtVs from AmpFire assays when making them as the reference for triage of HPV positive women through head-to-head comparison with the relevant CtVs from Cobas4800 HPV assay.

## Methods

### Study design and participants

This study was based on the data from a sub-study of The Chinese Multi-Center Screening Trial (CHIMUST) and its sub-study validating the effectiveness of AmpFire in detection of grade 2 and above cervical interepithelial neoplasia (CIN2 +).

CHIMUST (Registration number: ChiCTR-EOC-16008456) was a multicenter population-based cross-sectional cervical cancer screening study led by our team. Ten thousand eight hundred and eight-five (10,885) women from 15 screening sites in 7 provinces in China were screened from August 2016 to January 2018. Enrolled women was those who were aged 30–59 of years, sexually exposed, non-pregnant, unscreened for at least 3 years, without history of hysterectomy or pelvic radiation, and consent in writing for participation. The trial was approved by the Ethics Committee of the Peking University Shenzhen Hospital (IRB:PUSH2016001) and the Institutional Review Board of Cleveland Clinic (USA) (IRB:15–1549) [21].

Each participant provided a self-collected vaginal sample and a clinician-collected endocervical sample. The samples were split and tested for HPV using Cobas4800 (Roche, USA) and SeqHPV (BGI, Shenzhen, China). In addition, the clinician-collected samples were processed for cytology testing using ThinPrep (Hologic) [21].

Patients who tested positive for HPV on either assay (Cobas4800 and/or SeqHPV for self- and/or clinician-collected samples) were recalled for colposcopy, and colposcopy-directed and/or random biopsies were taken according to the Preventive Oncology International (POI) protocol [22]. All histology slides were analyzed by a gynecological pathology expert from PUSH(C.W.), who was blind of the HPV and cytology results. Histology results were classified as non-CIN, cervical intraepithelial neoplasia (CIN)1, CIN2, CIN3, adenocarcinoma in situ (AIS), and cancers.

The sub-study was conducted later, with aim to validate the performance of AmpFire in detection of CIN2 + and CIN3 + [20].The sub study was conducted using AmpFire to test the residual samples from 6,619 participants to from five locations: Pingxiang in Hebei Province (2,035), Huang Shi in Hubei Province (1,250), Chao Zhou in Guangdong Province (1,000), Mentougou in Beijing (988), and Xiang Huang Qi in Inner Mongolia (1,346) [20]. Before AmpFire testing, all the residual samples were stored at 4 °C.

### Cobas4800 HPV assay

The Cobas4800 HPV (hereafter as Cobas) is a multi-PCR-based HPV assay that detects a total of 14 hrHPV types across three channels: HPV16, HPV18, and a pooled 12-HPV channel (including HPV31, 33, 35, 39, 45, 51, 52, 56, 58, 59, 66, and 68), along with a separate β-globin channel as a reference. The manufacturers have defined CtV-cutoffs for all the HPV channels to determine positive results, which are 40.5 for HPV16 and 40 for HPV18 and the pooled 12-HPV channels. When the CtV is equal to or lower than the cutoff, a positive result is obtained and the corresponding CtV is recorded. The CtV of negative channels is above the default CtV-cutoff and is not reported.

### AmpFire HPV assay

The AmpFire assay (Atila BioSystems, Inc., CA, USA) is an isothermal, real-time fluorescent, multiplex nucleic acid amplification method which has received regulatory approval from both the European Community and the Chinese Food and Drug Administration. The AmpFire Multiplex HPV assay is able to detect the presence of 15 high-risk HPV genotypes (HPV16, 18, 31, 33, 35, 39, 45, 51, 52, 53, 56, 58, 59, 66, and 68) and can simultaneously genotype HPV16 and 18 separately in a single reaction. As same as in Cobas, it detects the human β-globin gene as an inner control. This assay can directly detect HPV from non-processed samples with limited hands-on time and no need for DNA extraction and purification. The amplification reaction is incubated at 60 °C for 75 min, and fluorescence is recorded every minute from each channel (HPV16, HPV18, non-16/18 hrHPV, β-globin). The Ct values for each amplification curve in all fluorescence channels are automatically reported. As the AmpFire assay is isothermal, there are no cycles, and the Ct value simply represents the reaction time in minutes. If the amplification curve for the β-globin gene is not exponential, the result is considered invalid [19].

In order to make the results from the AmpFire to be match with those from Cobas, we just included the cases those were: 1) tested positive of hr-HPV on Cobas4800 for the clinician-collected samples in the primary screening; 2) tested positive of hrHPV on AmpFire assays for clinician-collected samples in the sub-study; 3) with the CtV reported, and 4) with matched results from Cobas and Ampfire in terms of at least one HPV genotype.

When any case was reported by both assays to be the positive of non-16/18 hrHPV, the result from SeqHPV (that reports sequencing genotyped 14 hrHPV [16, 18, 31, 33, 35, 39, 45, 51, 52, 56, 58, 59, 66, and 68] individually) will be referred to determine the exact types those were reported by Cobas and AmpFire in pool. Cases would be excluded if 1) the HPV type of a single-type HPV was not matched on Cobas and AmpFire; 2) if the non-16/18 hrHPV from Cobas and AmpFire were reported by SeqHPV having HPV16 and/or 18; and 3) if the non-16/18 hrHPV from Cobas and AmpFire were reported multi-type infections by SeqHPV. (in such cases we could not identify which type of hrHPV was the determinant of the CtV for non-16/18 hrHPV). With the above inclusive and exclusive criteria, cases that were positive of HPV53 only on AmpFire were not included and had no influence on the analysis.

Cases with positive results for hrHPV from both the two assays with matched genotype information and identifiable genotype-related CtVs were included into the analysis (the analytic cases). The analytic cases were classified into three groups in a hierarchy manner per the genotypes: HPV16, HPV18, and non-16/18 hrHPV. If not specifically indicated, HPV16 referred to a result that was positive for HPV16 only or multiple HPV types including HPV16; HPV18 referred to a result that was positive for HPV18 only or multiple types including HPV18 but excluding HPV16; while non-16/18 hrHPV refers to a result that was positive of single hrHPV type that are none or excluding HPV16 and/or 18. CtV analysis was also conducted through categorizing the non-16/18 hrHPV according the genotyping from SeqHPV, into three groups: HPV A9 group (including HPV31, 33, 35, 52, 58), HPV A7 group (including HPV39, 45, 59, 68), and HPV A5/A6 group (including HPV51, 56, 66).

### Statistical analysis

The mean ± standard deviation was used to describe Ct values. Spearman's correlation coefficient was used to compare the association between CtV of hrHPV, HPV16, HPV18, non-16/18 hrHPV, and HPV A5/A6, A7, A9 group from the AmpFire and Cobas4800 assay. Spearman's correlation coefficient was also used to analyze the correlation between CtV from Cobas4800 or AmpFire and cervical lesion grades. Differences in CtV from Cobas4800 or AmpFire assay among histological lesion grades were compared by one-way ANOVA.

To differentiate low-CtV positives from high-CtV positives, the CtV cutoff was determined primarily based on the 4% CIN3 + incident rate [18], receiver operating characteristic curves (ROC curves) for predicting CIN3 + from CtV, and the quartile of the CtV of hrHPV, non-16/18 hrHPV, and A9 group. The cutoffs with good classification effect for positive women in both Cobas4800 and AmpFire assay were used to construct triage algorithms. McNemar's test was used to analyze the differences in sensitivity and specificity for detecting CIN2 + and CIN3 + in different triage algorithms.

Data analysis was performed using SPSS v.26.0 software (IBM, Armonk, NY, USA), and *P* < 0.05 was considered statistically significant.

## Results

A total of 6,042 women were included in the sub-study of CHIMUST and had HPV test results from both Cobas and AmpFire with complete data on cytology and histology analysis [20], of whom, 560 women had matched HPV positive results from both the tests and eligible for this analysis (the eligible cases). Among the 560 eligible cases, 99 cases were positive for HPV16, 31 for HPV18, and 430 for non-16/18 hrHPV. Of the cases positive of non-16/18 hrHPV, 165 were categorized into A9 group (HPV31, 33, 35, 52, 58), 90 into A7 group (HPV39, 45, 59, 68), and 61 into A5/A6 group (HPV51, 56, 66), 114 were excluded for positive of multi-type on SeqHPV without single-type corresponding CtV identifiable. Finally included in the analysis were 446 cases with matched HPV results and type-specific CtVs from both the analytic testing assays.

The mean age of the 446 women in the analytic group was 45.2 (± 7.42) years. Twenty-two-point twenty percent (22.20%, 99/446) of them were positive for HPV16, 6.95% (31/446) were positive for HPV18, and 70.85% (316/446) were positive for non-HPV16/18 hrHPV(Table 1). The CtV of hrHPV tested by Cobas4800 and AmpFire showed a linear correlation, with Spearman's correlation coefficients of 0.664, 0.818, 0.766, 0.749, 0.660, 0.815, and 0.775 for CtV of hrHPV, HPV16, HPV18, non-16/18 hrHPV, A5/A6 group, A7 group, and A9 group, respectively, with a significance level of *p* < 0.001 (Table 1).

**Table 1:** Consistency of Ct values from Cobas4800 and AmpFire assay

HPV genotypes	Cases(%)	Cobas4800 CtV	AmpFire CtV	Spearman	*p* value
hrHPV	446	30.16 ± 5.43	24.43 ± 8.17	0.664	< 0.001
HPV16	99(22.20)	29.42 ± 4.95	17.39 ± 4.08	0.818	< 0.001
HPV18	31(6.95)	31.92 ± 4.88	21.45 ± 6.27	0.766	< 0.001
Non-16/18 hrHPV	316(70.85)	30.22 ± 5.60	26.93 ± 7.91	0.749	< 0.001
A5/A6	61(13.68)	29.67 ± 5.12	24.33 ± 6.93	0.660	< 0.001
A7	90(20.18)	28.59 ± 5.52	27.59 ± 8.11	0.815	< 0.001
A9	165(37.00)	31.31 ± 5.60	27.53 ± 7.99	0.775	< 0.001

Pathologically, 66.82% (298/446) of the hrHPV positives were reported as non-CIN, 16.14% (72/446) as CIN1, 17.04% (76/446) as CIN2 + (including CIN2, CIN3, AIS, and cancers), and 7.26% (34/446) as CIN3 + (including CIN3, AIS, and cancers) (Supplementary Table1).

Our data revealed an inverse association between the severity of cervical lesions and the CtV of hrHPV, HPV16, non-16/18 hrHPV, and A9 group from both Cobas4800 and AmpFire assays. The Spearman's correlation coefficients were -0.278, -0.467, -0.23, and -0.327, respectively, between the CtV of hrHPV, HPV16, non-16/18 hrHPV, A9 group from Cobas4800 assay, and the severity of cervical lesions, with a significance level of *p* < 0.001. Similarly, the Spearman's correlation coefficients were -0.34, -0.344, -0.209, and -0.307, respectively, between the CtV from AmpFire and cervical lesion grades, with a significance level of *p* < 0.001.

**Fig. 1:**
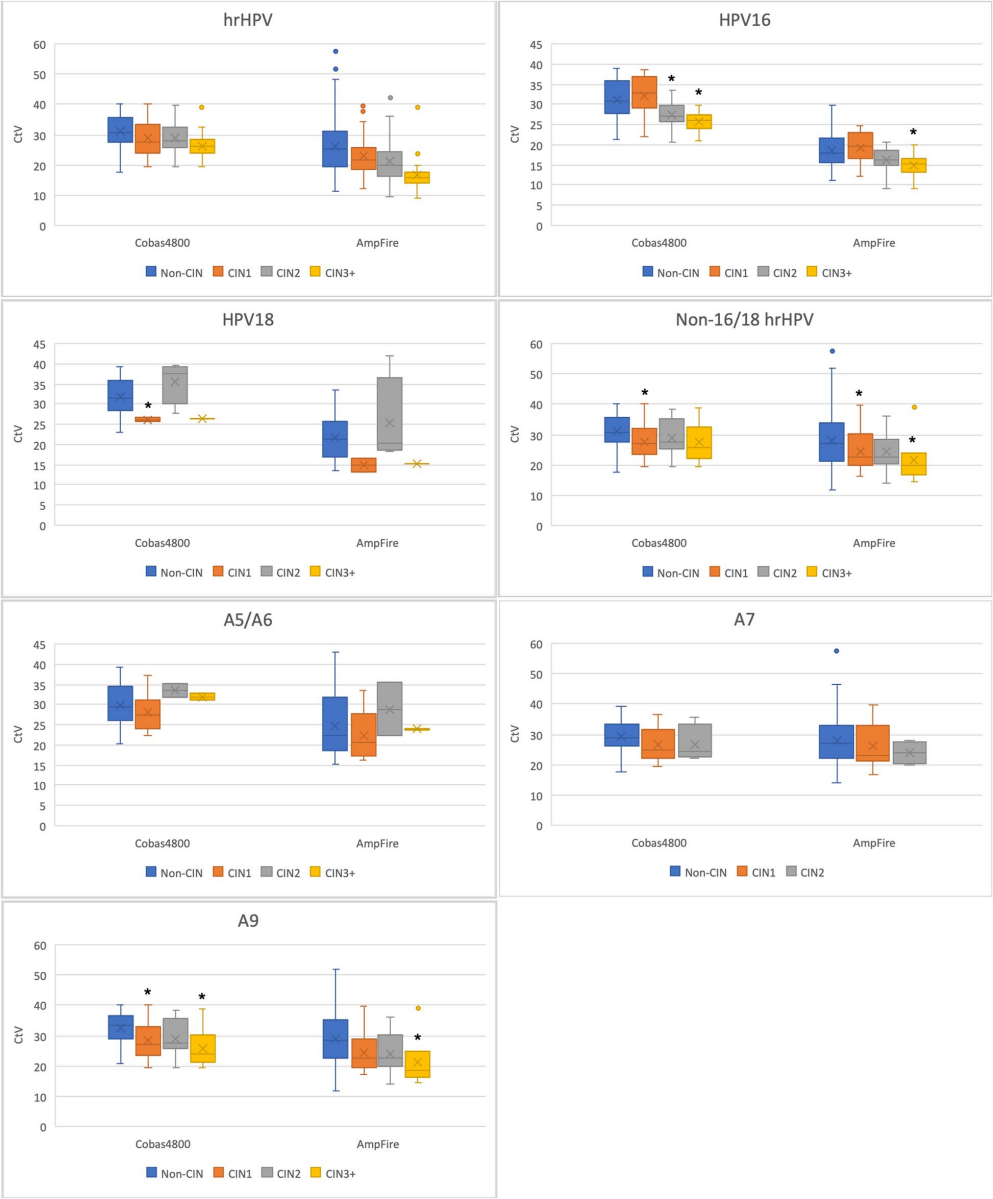
The HPV genotype specific CtVs of different grades of cervical lesions. CIN3 + refers to CIN3, AIS, and cancers. ^*^ *p* < 0.05 when compared with non-CIN

In both Cobas4800 and AmpFire assays, the CtVs of hrHPV in the CIN2 + and CIN3 + groups were significantly lower than those in the non-CIN group (*p* < 0.05). The CtVs of HPV16 in the CIN2 + and CIN3 + groups were significantly lower than those in both the non-CIN and CIN1 groups (*p* < 0.05). The CtVs of non-16/18 hrHPV in the CIN1 group were lower than those in the non-CIN group (*p* < 0.05), and the CtVs of A9 group in the CIN1 and CIN2 + cases were lower than those in the non-CIN cases (*p* < 0.05). No significant difference was found among different grades of lesion in terms of correlated CtVs of HPV18, A5/A6 group, and A7 group (Fig. 1, Supplementary Table1).

When we adopted the 50th percentile of CtV as the cutoff, we got a relatively satisfied differentiation of the women in hrHPV, non-16/18 hrHPV, and A9 HPV positive groups with high-CtVs from those with low-CtVs for Cobas4800 and AmpFire assays. With this cutoff, 91.18% of CIN3 + cases in hrHPV group were detected “low-CtV” on both Cobas4800 (CtV_Cobas_ ≤ 29.8) and AmpFire (CtV_AmpFire_ ≤ 22.6), 62.5% and 87.5% of CIN3 + cases in non-16/18 hrHPV group were detected “low-CtV” on Cobas4800 (CtV_Cobas_ ≤ 30) and AmpFire assay (CtV_AmpFire_ ≤ 25.71), respectively, and 83.33% of CIN3 + cases in A9 group were detected the low-CtV on both Cobas (CtV_Cobas_ ≤ 32) and AmpFire (CtV_AmpFire_ ≤ 26.48) (Table 2).

**Table 2:** PPV for CIN2 + and CIN3 + in low-Ct HPV positive and high-Ct HPV positive women grouped by 50% percentile of CtV (*n* = 446)

Cobas4800				AmpFire			
**Cutoff**	**Cases**	**CIN2 + **	**CIN3 + **	**Cutoff**	**Cases**	**CIN2 + **	**CIN3 + **
**hrHPV**	446	76	34	**hrHPV**	446	76	34
> 29.8	221(49.55)	20(26.32)	3(8.82)	> 22.6	222(49.78)	15(19.74)	3(8.82)
≤ 29.8	225(50.45)	56(73.68)	31(91.18)	≤ 22.6	224(50.22)	61(80.26)	31(91.18)
**HPV16**	99	41	25	**HPV16**	99	41	25
> 29.0	49(49.49)	9(21.95)	5(20.00)	> 16.0	49(49.49)	12(29.27)	6(24.00)
≤ 29.0	50(50.51)	32(78.05)	20(80.00)	≤ 16.0	50(50.51)	29(70.73)	19(76.00)
**HPV18**	31	5	1	**HPV18**	31	5	1
> 31.6	15(48.39)	3(60.00)	0(0.00)	> 20.1	15(48.39)	2(40.00)	0(0.00)
≤ 31.6	16(51.61)	2(40.00)	1(100.00)	≤ 20.1	16(51.61)	3(60.00)	1(100.00)
**Non-16/18 hrHPV**	316	30	8	**Non-16/18 hrHPV**	316	30	8
> 30	157(49.68)	13(43.33)	3(37.5)	> 25.71	158(50)	9(30)	1(12.5)
≤ 30	159(50.32)	17(56.67)	5(62.5)	≤ 25.71	158(50)	21(70)	7(87.5)
**A9**	165	22	6	**A9**	165	22	6
> 32	81(49.09)	5(22.73)	1(16.67)	> 26.48	82(49.7)	6(27.27)	1(16.67)
≤ 32	84(50.91)	17(77.27)	5(83.33)	≤ 26.48	83(50.3)	16(72.73)	5(83.33)

Using the 50th percentile of CtV as a cutoff, we developed triage algorithms for HPV-positive patients in different groups and compared them with the recommended algorithms (Table 3). Compared with the commonly accepted algorithm of “HPV16/18 genotypes plus cytology ≥ ASCUS for non-16/18 hrHPV” (algorithm D), using “genotype for HPV16/18 plus low-CtV for non-16/18 hrHPV” to triage all women positive of hrHPV (algorithm E/F) could yield a comparable sensitivity of 91.9% in both Cobas and AmpFire and a specificity of 52.91% in Cobas4800 and 53.16% in AmpFire assay for CIN3 + , with the colposcopy referral rate of 50.45% and 50.22%, respectively. When triage the the positive women using “genotypes for HPV16/18 and low-CtV for non-16/18 hrHPV” (algorithm G/H), a comparable sensitivity of 91.18% in Cobas4800 and 97.06% in AmpFire assay for CIN3 + can be achieved but the specificity was too low to the acceptance (37.38% in Cobas and 38.11% in AmpFire assay), and the colposcopy referrals was rated to be 64.80% and 64.57%, respectively. However, when using "genotype for HPV16/18 plus low-CtV for A9 group” (algorithm I/J), a comparable sensitivity of 91.18% in both Cobas and AmpFire and a higher specificity of 55.58% in Cobas and 55.83% in AmpFire can be achieved for CIN3 + , with the colposcopy referral rates of 47.98% and 47.76%, respectively. (Table 3).

**Table 3:** Comparison of different triage algorithms (*n* = 446)

Algorithms	CIN2 +		CIN3 +		Colposcopy referral rate%	Cytology testing rate%	Colposcopies to detect 1 CIN2 + /CIN3 +
	Sen%	Spe%	Sen%	Spe%			
A. HPV16 /18	60.53^*^	77.30^*^	76.47^*^	74.76^*^	29.15	0	2.83/5.00
B. Cytology ≥ ASCUS	78.95^*^	68.11^*^	100	65.05^*^	39.91	100	2.97/5.24
C. Cytology ≥ LSIL	65.79^*^	82.16^*^	94.12	79.61^*^	26.01	100	2.32/3.63
D. HPV16/18 + cytology ≥ ASCUS for non-16/18 hrHPV	90.79	52.70	100	49.03	54.71	70.85	3.54/7.18
E. Low-CtV for HrHPV (Cobas)	73.68^*^	54.32	91.18	52.91	50.45	0	4.02/7.26
F. Low-CtV for HrHPV (AmpFire)	80.26^*^	55.95	91.18	53.16	50.22	0	3.67/7.23
G. HPV16 /18 + low-CtV for non-16/18 hrHPV (Cobas)	82.89	38.92^*^	91.18	37.38^*^	64.80	0	4.59/9.32
H. HPV16 /18 + low-CtV for non-16/18 hrHPV (AmpFire)	88.16	40.27^*^	97.06	38.11^*^	64.57	0	4.30/8.73
I. HPV16/18 + low-CtV for A9 (Cobas)	82.89	59.19^*^	91.18	55.58^*^	47.98	0	3.40/6.90
J. HPV16/18 + low-CtV for A9 (AmpFire)	81.58	59.19^*^	91.18	55.83^*^	47.76	0	3.44/6.87

^*^Compared with algorithm D, *p* < 0.05

## Discussion

### The advantages of AmpFire HPV assay in cervical cancer screening

Low-and-middle-income countries (LMICs) bear a disproportionate burden of the world cervical cancer incidence and mortality. Control of cervical cancer in LMICs presents unique challenges those are differ from high-income countries (HICs). According to a statistic report in 2020, less developed regions accounted for 88.1% of cervical cancer incidence and 91.4% of mortality [1]. However, in other hand, the screening coverage of eligible women in most LMICs was just 19%, compared to 63% in HICs [23].

The AmpFire assay has proven to be equally accurate to Cobas4800 and SeqHPV in detection of CIN2 + or CIN3 + for both self- and clinician-collected samples [20]. However, AmpFire in advantageous of the unique isothermal multiplex nucleic acid amplification technology that does not require a special PCR lab or air flow management, and therefore enables the tests be conducted directly on self- or clinician-collected the samples on the screening site, without need to process and preserve the samples with any liquid. Due to the above advantage, Ampfire is compatible with inexpensive and portable equipment. These features make it a promising assay, especially in LMICs. AmpFire assay can report the HPV testing results in less than 1.5 h [24], which enables the “screen and treat” cervical screening programs be designed on self-collected HPV testing and completed within one day [20], the recognized solution for increasing the coverage of screening and the rate of precancer treatment. However, here comes a question, what is the appreciated triage in the “screen and treat” program when us HPV testing as the primary screening?

### The potentiality of triaging hrHPV positive women with CtVs

Self-sampling has been proven to be an effective way to increase screening coverage in LMICs [25, 26]. However, it is a unique challenge to opt applicable and effective triages in these settings after primary screening with self-collected HPV testing. Triage protocols based on cytology could not work due to the shortage of cytologists and the lack of quality assurance mechanisms in LMICs. Moreover, cytology does not work with self-collected samples, therefore additional clinic visits for just triage will be requires for the women who are primarily tested positive of hrHPV, which would increase the loss to follow-up [5]. Triage protocols based molecular test on self-collected samples is obviously the right option for management of positives because it is an objective reference, less dependent to cytology resources, more affordable to the community, and beneficial to positive management by minimizing the requirements for clinic visits.

In addition of HPV genotypes, HPV viral load from Cobas have been demonstrated to be a potential triage option [16–18]. Given the dominant proportion of HPV16/18 infections in cervical cancer cases and the increased risk of high-grade cervical lesions among HPV16/18-positive women, referring all HPV16/18-positive women for colposcopy is supported by guidelines [6]. However, although the sensitivity for detecting CIN2 + and CIN3 + can be rated to be 49.3% and 68.5%, respectively, with that approach [17], triage of the women positive of other hrHPV types is progressively necessary along with the use of HPV vaccines that will reduced the risk of HPV16/18 associated CIN2 + and CIN3 + [27]. Therefore, optimizing the management of women infected with non-16/18 hrHPV is becoming increasingly important to cervical cancer control [28].

This analysis demonstrated the correlation between the CtVs of HPV16 and non-16/18 hrHPV and the severity of cervical lesions are reverse on both the Cobas4800 and AmpFire assays. Notably, when using Spearman correlation coefficient to express the relativity of the CtV and the severity of cervical lesions, the relativity of HPV-16 CtV with lesion severity is stronger than that of the CtV of non-16/18 hrHPV, which was consistent with prior analyses based on CHIMUST [16–18]. The non-16/18 hrHPV were demonstrated vary in risks for cervical precancerous lesions [29–31]. Those genotypes were grouped into A5/A6, A7, and A9 groups according to their evolutionary relationships. HPV types in the A9 group (HPV31, 33, 35, 52, 58) was phylogenetically similar to HPV16, those in the A7 group (HPV39, 45, 59, and 68) was similar to HPV18, and those in the A5/A6 group (HPV51, 56, 66) was not similar to either HPV16 or HPV18. Our results indicated that the CtVs of the A9 group was inversely correlated with the severity of cervical lesions, with a correlation that is stronger than the CtVs of non-16/18 hrHPV. No significant relationship was found between the CtVs of A7 and A5/A6 groups and the severity of the cervical lesions. These findings are consistent with prior studies, such as Adcock et al*.*, who reported that the top three HPV genotypes (HPV16/33/31) with the highest CIN2 + /CIN3 + risk were all within the A9 group [29]. Similarly, Li et al*.*found that the viral load of some hrHPV genotypes (HPV 16, 18, 31, 33, 51, 52, 53, and 58) were positively correlated with the severity of cervical lesions but others were not [30]. Duan et al*.*also observed significant differences in viral loads of HPV16, 33, and 58 among different grades of cervical lesions, but no difference was observed in other genotypes [31].

Compared to the A5/A6 and A7 groups, the A9 group was proofed a higher prevalence and a higher risk of CIN2 + /CIN3 + . Our findings indicate that the A9 group that proportioned 52.2% (165/316) of the non-16/18 hrHPV types accounted for 73.3% (22/30) of the CIN2 + and 75% (6/8) of the CIN3 + cases resulted by the non-16/18 hrHPV. This is consistent with results from large population-based studies, such as Zhu et al*.*who analyzed data from 1.7 million women across 68 population-based studies and showed that HPV16, 52, 58, 18, and 33 were the most prevalent subtypes in China [32]. Furthermore, in a study conducted by Du et al*.*, the pooled data from 3045 women who tested positive for hrHPV with self-collected samples revealed that the three most prevalent genotypes were HPV52, HPV16, and HPV58. CIN2 + was found most frequently in HPV16 (31.23%), HPV33 (24.03%), HPV58 (18.41%), HPV31 (11.76%), HPV18 (7.75%), and HPV52 (7.30%) [33].

Noting the high prevalence of the A9 group in the Chinese population, our team in the prior work evaluated algorithms which use the A9 group from Cervista Assay (Cervista A9) for primary cervical cancer screening, and found it similarly sensitive for detection of CIN2 + and CIN3 + when compared to the full Cervista assay, but significantly reduced the colposcopy referral rate and cytology rate [34]. In this study, our results showed that, comparing with the triage algorithm G/H (HPV16/18 + low-CtV for non-16/18 hrHPV), the triage algorithm I/J (HPV16/18 + low-CtV for A9 group) showed comparable sensitivity but significantly higher specificity for detecting CIN2 + and CIN3 + . The latter also showed comparable sensitivity and significantly higher specificity for detecting CIN2 + and CIN3 + compared with the guideline-recommended algorithm of HPV16/18 + cytology ≥ ASCUS for non-HPV16/18 types). These results suggest that using high viral load (or low CtVs) as reference to triage A9 group is satisfied in sensitivity and specificity for detecting CIN2 + /CIN3 + , which downrates the colposcopy referrals and makes cytology unnecessary. Triage with virus load or CtV is a promising approach that could potentially improve cervical cancer screening in areas with high A9 group prevalence. Most importantly, our study highlights the utility of Ct value as a good triage marker in both PCR-based and isothermal amplification HPV detection.

### Advantages and limitations

Our study evaluated data collected from a well-organized, population-based cervical screening program that involved 446 HPV-positive women from five provinces in China. Cervical exfoliated cell samples were obtained using a standardized sampling procedure, which makes the results applicable to the general population in various settings.

However, the study also has limits. Firstly, although our results showed no significant difference in the sensitivity for detecting CIN2 + and CIN3 + between the triage algorithm combining HPV genotypes with viral load (triage algorithm G/H/I/J) and that combining HPV genotypes with cytology, the guideline-recommended algorithm, we cannot ignore the fact that part of the CIN3 + showed relative higher CtV, which could cause case missing when we adopt the genotype plus virus-load algorithm. In our study, there were 3 CIN3 + cases tested positive of non-16/18 hrHPV on Cobas HPV with high CtV. Among them one was reported high CtV by AmpFire HPV test as well. Some studies described that HPV integration into the genome of the cells were observed particularly in CIN3 and cervical cancer [35]. Complete integration might hide the detectable biomarkers of HPV and could be a problem to detection of HPV based on biomarker of DNA. This would provide a drawback in using HPV viral load as a triage parameter. Introduction of biomarkers related to HPV integration to human genome like p16 cytology [36] or E6 and E7 (E6/E7)mRNA [37, 38] may help to improve the sensitivity, the cost should also be considered to get the best benefit [38]. Another weakness of our analysis is the number of CIN3 + cases in the analytic cases. Thirty-four (34) CIN3 cases with only 8 positive of non-16/18 hrHPV may not power enough in terms of statistics. In addition, due to technical limitation, we could not differ the CtV of each specific genotype in a multi-genotype infections, and cases with multi-genotype non-16/18 hrHPV were excluded from the analysis, which might weaken the representativeness in multi-genotype non-16/18 hrHPV positive women.

## Conclusions

In conclusion, the HPV viral loads reflected by CtVs for hrHPV, HPV16, non-16/18 hrHPV types, and A9 group were linearly correlated with the severity of cervical intraepithelial neoplasia in both Cobas and AmpFire. In comparison with the algorithm of “Genotype for HPV16/18 combined with Cytology for non-16/18 hrHPV types”, the algorithms of “Genotype for HPV16/18 combined with low-CtV for non-16/18 hrHPV” from either Cobas or AmpFire is equal sensitive but less specific in detection of CIN2 + and CIN3 + , and the algorithm of “Genotype for HPV16/18 combined with low-CtV for A9 group” is equal sensitive but significantly higher specific in detection of CIN2 + or CIN3 + . Both of the algorithms are satisfied enough to be a triage in cervical cancer screening services in lower-resource area where qualified cytology is not available.

### Supplementary Information

**Additional file 1: Supplementary table 1.** Ct values for specific HPV genotype among different grades of cervical lesions. **Supplementary table 2.** Distribution of CIN2+ and CIN3+ in low-Ct HPV positive and high-Ct HPV positive women according to different classification methods (*n*=446)

## Data Availability

The datasets generated and/or analyzed during the current study are not publicly available but are available from the corresponding author on reasonable request.

## References

[1] Sung H (2021). Global Cancer Statistics 2020: GLOBOCAN Estimates of Incidence and Mortality Worldwide for 36 Cancers in 185 Countries. CA Cancer J Clin.

[2] Schiffman M, Castle PE, Jeronimo J, Rodriguez AC, Wacholder S (2007). Human papillomavirus and cervical cancer. Lancet.

[3] Ronco G (2014). Efficacy of HPV-based screening for prevention of invasive cervical cancer: follow-up of four European randomised controlled trials. Lancet.

[4] Arbyn M (2010). European guidelines for quality assurance in cervical cancer screening. Second edition summary document. Ann Oncol..

[5] Cuschieri K (2018). Eurogin roadmap 2017: Triage strategies for the management of HPV-positive women in cervical screening programs. Int J Cancer.

[6] Huh WK (2015). Use of primary high-risk human papillomavirus testing for cervical cancer screening: interim clinical guidance. Gynecol Oncol.

[7] Sørbye SW (2017). Accuracy of cervical cytology: comparison of diagnoses of 100 Pap smears read by four pathologists at three hospitals in Norway. BMC Clin Pathol.

[8] Schiffman M (2015). A study of genotyping for management of human papillomavirus-positive, cytology-negative cervical screening results. J Clin Microbiol.

[9] Luo H (2017). Evaluation of viral load as a triage strategy with primary high-risk human papillomavirus cervical cancer screening. J Low Genit Tract Dis.

[10] Long W (2018). HPV-16, HPV-58, and HPV-33 are the most carcinogenic HPV genotypes in Southwestern China and their viral loads are associated with severity of premalignant lesions in the cervix. Virol J.

[11] Li TY (2018). Association between high risk human papillomavirus DNA load and cervical lesions in different infection status. Zhonghua Zhong Liu Za Zhi.

[12] Xu L, Oštrbenk Valenčak A, Poljak M, Arbyn M. Evaluation and Optimization of the Clinical Accuracy of Hybribio's 14 High-Risk HPV with 16/18 Genotyping Assay within the VALGENT-3 Framework. J Clin Microbiol. 2020;58, 10.1128/jcm.00234-20. 10.1128/JCM.00234-20 PMC7269387 32245832

[13] Kuhn L (2020). Clinical evaluation of modifications to a human papillomavirus assay to optimise its utility for cervical cancer screening in low-resource settings: a diagnostic accuracy study. Lancet Glob Health.

[14] Álvarez-Argüelles ME (2015). Quantification of human papilloma virus (HPV) DNA using the Cobas 4800 system in women with and without pathological alterations attributable to the virus. J Virol Methods.

[15] Jazmati N, Hellmich M, Ličanin B, Plum G, Kaasch AJ (2016). PCR cycle threshold value predicts the course of Clostridium difficile infection. Clin Microbiol Infect.

[16] Duan L (2020). The effectiveness of HPV viral load, reflected by Cobas 4800 HPV-Ct values for the triage of HPV-positive women in primary cervical cancer screening: Direct endocervical samples. PLoS ONE.

[17] Song F (2021). The effectiveness of human papillomavirus load, reflected by cycle threshold values, for the triage of HPV-positive self-samples in cervical cancer screening. J Med Screen.

[18] Zhang Y (2022). Verification of the association of the cycle threshold (Ct) values from HPV testing on Cobas4800 with the histologic grades of cervical lesions using data from two population-based cervical cancer screening trials. Infect Agent Cancer.

[19] Tang YW (2020). An Isothermal, multiplex amplification assay for detection and genotyping of human papillomaviruses in formalin-fixed. Paraffin-Embedded Tissues J Mol Diagn.

[20] Zhang W (2020). Evaluation of an isothermal amplification HPV detection assay for primary cervical cancer screening. Infect Agent Cancer.

[21] Du H (2021). Evaluation of Cobas HPV and SeqHPV assays in the chinese multicenter screening trial. J Low Genit Tract Dis.

[22] Belinson JL, Pretorius RG (2016). A standard protocol for the colposcopy exam. J Low Genit Tract Dis.

[23] Gakidou E, Nordhagen S, Obermeyer Z (2008). Coverage of cervical cancer screening in 57 countries: low average levels and large inequalities. PLoS Med.

[24] Jang D (2020). Performance of AmpFire HPV assay on neck cervical lymph node aspirate and oropharyngeal samples. J Virol Methods.

[25] Fargnoli V, Petignat P, Burton-Jeangros C (2015). To what extent will women accept HPV self-sampling for cervical cancer screening? A qualitative study conducted in Switzerland. Int J Womens Health.

[26] Inturrisi F (2021). Clinical performance of high-risk HPV testing on self-samples versus clinician samples in routine primary HPV screening in the Netherlands: an observational study. Lancet Reg Health Eur.

[27] Arbyn M, Xu L, Simoens C, Martin-Hirsch PP (2018). Prophylactic vaccination against human papillomaviruses to prevent cervical cancer and its precursors. Cochrane Database Syst Rev.

[28] Dong L (2018). Human papillomavirus viral load as a useful triage tool for non-16/18 high-risk human papillomavirus positive women: a prospective screening cohort study. Gynecol Oncol.

[29] Adcock R, Cuzick J, Hunt WC, McDonald RM, Wheeler CM (2019). Role of HPV Genotype, Multiple Infections, and Viral Load on the Risk of High-Grade Cervical Neoplasia. Cancer Epidemiol Biomarkers Prev.

[30] Li Y (2021). Correlation between multi-type human papillomavirus infections and viral loads and the cervical pathological grade. Int J Gynaecol Obstet.

[31] Duan L (2020). The application of BMRT-HPV viral load to secondary screening strategies for cervical cancer. PLoS One.

[32] Zhu B (2019). The prevalence, trends, and geographical distribution of human papillomavirus infection in China: The pooled analysis of 1.7 million women. Cancer Med..

[33] Du H (2021). The prevalence of HR-HPV infection based on self-sampling among women in China exhibited some unique epidemiologic features. J Clin Epidemiol.

[34] Zhao J (2016). Evaluation of The Cervista HPV A9 group In Screening Patients for Cervical Cancer. J Med Screen.

[35] Oyervides-Muñoz MA (2018). Understanding the HPV integration and its progression to cervical cancer. Infect Genet Evol.

[36] Song F (2021). Evaluation of p16(INK4a) immunocytology and human papillomavirus (HPV) genotyping triage after primary HPV cervical cancer screening on self-samples in China. Gynecol Oncol.

[37] Giorgi Rossi P (2021). p16/ki67 and E6/E7 mRNA Accuracy and Prognostic Value in Triaging HPV DNA-Positive Women. J Natl Cancer Inst.

[38] Derbie A, Mekonnen D, Woldeamanuel Y, Van Ostade X, Abebe T (2020). HPV E6/E7 mRNA test for the detection of high grade cervical intraepithelial neoplasia (CIN2+): a systematic review. Infect Agent Cancer.

