# Supplementary material for: A comparative analysis of cycle threshold (Ct) values from Cobas4800 and AmpFire HPV assay for triage of women with positive hrHPV results
Source: BMC Infect Dis. 2023 Nov 10;23:783. doi: 10.1186/s12879-023-08737-4 (PMC10636838; doi:10.1186/s12879-023-08737-4)
Supplement: Supplementary file 1 — Additional file 1: Supplementary table 1. Ct values for specific HPV genotype among different grades of cervical lesions. Supplementary table 2. Distribution of CIN2+ and CIN3+ in low-Ct HPV positive and high-Ct HPV positive women according to different classification methods (n=446) [file 12879_2023_8737_MOESM1_ESM.docx]

**Supplementary table 1.** Ct values for specific HPV genotype among different grades of cervical lesions

|  | **Cases (%)** | **Cobas4800 CtV** | **AmpFire CtV** |
| --- | --- | --- | --- |
| **HrHPV** | 446 | 30.16±5.43 | 24.43±8.17 |
| Non-CIN | 298(66.82) | 31.13±5.26 | 26.09±8.29 |
| CIN1 | 72(16.14) | 28.65±5.52^a^ | 23.1±6.43^a^ |
| CIN2 | 42(9.42) | 29.01±5.54 | 21.32±6.98^a^ |
| CIN3 | 31(6.95) | 26.25±3.85^a^ | 16.69±5.31^ab^ |
| AIS | 1(0.22) | 26.50 | 15.17 |
| Cancer | 2(0.45) | 26.25±4.74 | 14.85±2.30 |
| CIN2+ | 76(17.04) | 27.78±4.99^a^ | 19.18±6.62^ab^ |
| CIN3+ | 34(7.26) | 26.25±3.77^a^ | 16.53±5.1^ab^ |
| **HPV16** | 99 | 29.42±4.95 | 17.39±4.08 |
| Non-CIN | 42(42.42) | 31.31±5.02 | 18.61±4.49 |
| CIN1 | 16(16.16) | 32.04±5.06 | 19.33±3.87 |
| CIN2 | 16(16.16) | 27.34±3.52^ab^ | 16.16±2.87 |
| CIN3 | 23(23.23) | 25.86±2.46^ab^ | 14.91±2.63^ab^ |
| Cancer | 2(2.02) | 26.25±4.74 | 14.85±2.30 |
| CIN2+ | 41(41.41) | 26.45±3.01^ab^ | 15.40±2.72^ab^ |
| CIN3+ | 25(25.25) | 25.89±2.55^ab^ | 14.91±2.56^ab^ |
| **HPV18** | 31 | 31.92±4.88 | 21.45±6.27 |
| Non-CIN | 24(77.42) | 31.99±4.55 | 21.63±5.15 |
| CIN1 | 2(6.45) | 26.15±0.78^a^ | 14.93±2.44 |
| CIN2 | 4(12.90) | 35.70±5.33 | 25.25±11.24 |
| AIS | 1(3.23) | 26.5 | 15.17 |
| CIN2+ | 5(16.13) | 33.86±6.18 | 23.24±10.73 |
| CIN3+ | 1(3.23) | 26.5 | 15.17 |
| **Non-16/18 hrHPV** | 316 | 30.22±5.6 | 26.93±7.91 |
| Non-CIN | 232(73.42) | 31.01±5.39 | 27.91±8.16 |
| CIN1 | 54(17.09) | 27.74±5.38^a^ | 24.52±6.49^a^ |
| CIN2 | 22(6.96) | 29.01±6.03 | 24.36±6.20 |
| CIN3 | 8(2.53) | 27.36±6.54 | 21.79±7.68^a^ |
| CIN2+ | 30(9.49) | 28.57±6.1 | 23.67±6.59 |
| CIN3+ | 8(2.53) | 27.36±6.54 | 21.79±7.68^a^ |
| **A5/A6** | 61 | 29.67±5.12 | 24.33±6.93 |
| Non-CIN | 44(72.13) | 29.82±5.34 | 24.69±7.17 |
| CIN1 | 13(21.31) | 28.22±4.75 | 22.47±6.42 |
| CIN2 | 2(3.28) | 33.55±2.33 | 28.86±9.36 |
| CIN3 | 2(3.28) | 31.9±1.13 | 23.88±0.02 |
| CIN2+ | 4(6.56) | 32.73±1.77 | 26.37±6.12 |
| CIN3+ | 2(3.28) | 31.9±1.13 | 23.88±0.02 |
| **A7** | 90 | 28.59±5.52 | 27.59±8.11 |
| Non-CIN | 70(77.78) | 29.2±5.45 | 28.09±8.48 |
| CIN1 | 16(17.78) | 26.41±5.33 | 26.3±7.07 |
| CIN2 | 4(4.44) | 26.73±6.23 | 23.92±3.75 |
| **A9** | 165 | 31.31±5.6 | 27.53±7.99 |
| Non-CIN | 118(71.52) | 32.54±4.94 | 29±8.06 |
| CIN1 | 25(15.15) | 28.33±5.78^a^ | 24.44±6.07^a^ |
| CIN2 | 16(9.70) | 29.02±6.22 | 23.91±6.50 |
| CIN3 | 6(3.64) | 25.85±6.97^a^ | 21.09±8.95 |
| CIN2+ | 22(13.33) | 28.15±6.43^a^ | 23.14±7.13^a^ |
| CIN3+ | 6(3.64) | 25.85±6.97^a^ | 21.09±8.95 |

^a^compared with non-CIN, p<0.05.

^b^compared with CIN1, p<0.05.

**Supplementary table 2.** Distribution of CIN2+ and CIN3+ in low-Ct HPV positive and high-Ct HPV positive women according to different classification methods (n=446)

| **Cobas4800** | | | | | **AmpFire** | | | | |
| --- | --- | --- | --- | --- | --- | --- | --- | --- | --- |
| **Cutoff** | **Cases** | **CIN2+** | **CIN3+** | **Cutoff** | | **Cases** | **CIN2+** | **CIN3+** |  |
| **Cutoffs according to CtV related to the 4% CIN3+ incident rate** | | | | | | | | | |
| **hrHPV** | 446 | 76 | 34 | **hrHPV** | | 446 | 76 | 34 |  |
| >33.7 | 132(29.6) | 10(13.16) | 1(2.94) | >24.4 | | 191(42.83) | 11(14.47) | 1(2.94) |  |
| ≤33.7 | 314(70.4) | 66(86.84) | 33(97.06) | ≤24.4 | | 255(57.17) | 65(85.53) | 33(97.06) |  |
| **Non-16/18 hrHPV** | 316 | 30 | 8 | **Non-16/18 hrHPV** | | 316 | 30 | 8 |  |
| >28.4 | 192(60.76) | 13(43.33) | 3(37.5) | >25.63 | | 161(50.95) | 9(30) | 1(12.5) |  |
| ≤28.4 | 124(39.24) | 17(56.67) | 5(62.5) | ≤25.63 | | 155(49.05) | 21(70) | 7(87.5) |  |
| **A9** | 165 | 22 | 6 | **A9** | | 165 | 22 | 6 |  |
| >34.6 | 56(33.94) | 5(22.73) | 1(16.67) | >29.9 | | 59(35.76) | 5(22.73) | 1(16.67) |  |
| ≤34.6 | 109(66.06) | 17(77.27) | 5(83.33) | ≤29.9 | | 106(64.24) | 17(77.27) | 5(83.33) |  |
| **Cutoffs according to ROC curves** | | | | | | | | | |
| **hrHPV** | 446 | 76 | 34 | **hrHPV** | | 446 | 76 | 34 |  |
| >29.7 | 224(50.22) | 20(26.32) | 3(8.82) | >17.69 | | 342(76.68) | 35(46.05) | 7(20.59) |  |
| ≤29.7 | 222(49.78) | 56(73.68) | 31(91.18) | ≤17.69 | | 104(23.32) | 41(53.95) | 27(79.41) |  |
| **HPV16** | 99 | 41 | 25 | **HPV16** | | 99 | 41 | 25 |  |
| >29.7 | 41(41.41) | 4(9.76) | 0(0) | >16.63 | | 47(47.47) | 10(24.39) | 4(16) |  |
| ≤29.7 | 58(58.59) | 37(90.24) | 25(100) | ≤16.63 | | 52(52.53) | 31(75.61) | 21(84) |  |
| **HPV18** | 31 | 5 | 1 | **HPV18** | | 31 | 5 | 1 |  |
| >25.8 | 28(90.32) | 5(100) | 1(100) | >15.17 | | 27(87.10) | 4(80) | 0(0) |  |
| ≤25.8 | 3(9.68) | 0(0) | 0(0) | ≤15.17 | | 4(12.90) | 1(20) | 1(100) |  |
| **Non-16/18 hrHPV** | 316 | 30 | 8 | **Non-16/18 hrHPV** | | 316 | 30 | 8 |  |
| >23.7 | 266(84.18) | 21(70) | 4(50) | >24.89 | | 184(58.23) | 11(36.67) | 1(12.5) |  |
| ≤23.7 | 50(15.82) | 9(30) | 4(50) | ≤24.89 | | 132(41.77) | 19(63.33) | 7(87.5) |  |
| **A9** | 165 | 22 | 6 | **A9** | | 165 | 22 | 6 |  |
| >27.5 | 121(73.33) | 9(40.91) | 1(16.67) | >19.85 | | 133(80.61) | 13(59.09) | 1(16.67) |  |
| ≤27.5 | 44(26.67) | 13(59.09) | 5(83.33) | ≤19.85 | | 32(19.39) | 9(40.91) | 5(83.33) |  |
| **75% percentile of CtV as cutoffs** | | | | | | | | | |
| **hrHPV** | 446 | 76 | 34 | **hrHPV** | | 446 | 76 | 34 |  |
| >34.9 | 110(24.66) | 10(13.16) | 1(2.94) | >29.68 | | 111(24.89) | 7(9.21) | 1(2.94) |  |
| ≤34.9 | 336(75.34) | 66(86.84) | 33(97.06) | ≤29.68 | | 335(75.11) | 69(90.79) | 33(97.06) |  |
| **HPV16** | 99 | 41 | 25 | **HPV16** | | 99 | 41 | 25 |  |
| >32.9 | 24(24.24) | 1(2.44) | 0(0) | >19.52 | | 24(24.24) | 3(7.32) | 1(4) |  |
| ≤32.9 | 75(75.76) | 40(97.56) | 25(100) | ≤19.52 | | 75(75.76) | 38(92.68) | 24(96) |  |
| **HPV18** | 31 | 5 | 1 | **HPV18** | | 31 | 5 | 1 |  |
| >36.6 | 7(22.58) | 3(60.00) | 0(0.00) | >24.98 | | 7(22.58) | 1(20) | 0(0.00) |  |
| ≤36.6 | 24(77.42) | 2(40.00) | 1(100.00) | ≤24.98 | | 24(77.42) | 4(80) | 1(100.00) |  |
| **Non-16/18 hrHPV** | 316 | 30 | 8 | **Non-16/18 hrHPV** | | 316 | 30 | 8 |  |
| >35.2 | 75(23.73) | 6(20) | 1(12.5) | >32.45 | | 79(25) | 4(13.33) | 1(12.5) |  |
| ≤35.2 | 241(76.27) | 24(80) | 7(87.5) | ≤32.45 | | 237(75) | 26(86.67) | 7(87.5) |  |
| **A9** | 165 | 22 | 6 | **A9** | | 165 | 22 | 6 |  |
| >36.3 | 41(24.85) | 5(22.73) | 1(16.67) | >33.58 | | 41(24.85) | 3(13.64) | 1(16.67) |  |
| ≤36.3 | 124(75.15) | 17(77.27) | 5(83.33) | ≤33.58 | | 124(75.15) | 19(86.36) | 5(83.33) |  |
| **50% percentile of CtV as cutoffs** | | | | | | | | | |
| **hrHPV** | 446 | 76 | 34 | **hrHPV** | | 446 | 76 | 34 |  |
| >29.8 | 221(49.55) | 20(26.32) | 3(8.82) | >22.6 | | 222(49.78) | 15(19.74) | 3(8.82) |  |
| ≤29.8 | 225(50.45) | 56(73.68) | 31(91.18) | ≤22.6 | | 224(50.22) | 61(80.26) | 31(91.18) |  |
| **HPV16** | 99 | 41 | 25 | **HPV16** | | 99 | 41 | 25 |  |
| >29.0 | 49(49.49) | 9(21.95) | 5(20.00) | >16.0 | | 49(49.49) | 12(29.27) | 6(24.00) |  |
| ≤29.0 | 50(50.51) | 32(78.05) | 20(80.00) | ≤16.0 | | 50(50.51) | 29(70.73) | 19(76.00) |  |
| **HPV18** | 31 | 5 | 1 | **HPV18** | | 31 | 5 | 1 |  |
| >31.6 | 15(48.39) | 3(60.00) | 0(0.00) | >20.11 | | 15(48.39) | 2(40.00) | 0(0.00) |  |
| ≤31.6 | 16(51.61) | 2(40.00) | 1(100.00) | ≤20.11 | | 16(51.61) | 3(60.00) | 1(100.00) |  |
| **Non-16/18 hrHPV** | 316 | 30 | 8 | **Non-16/18 hrHPV** | | 316 | 30 | 8 |  |
| >30 | 157(49.68) | 13(43.33) | 3(37.5) | >25.71 | | 158(50) | 9(30) | 1(12.5) |  |
| ≤30 | 159(50.32) | 17(56.67) | 5(62.5) | ≤25.71 | | 158(50) | 21(70) | 7(87.5) |  |
| **A9** | 165 | 22 | 6 | **A9** | | 165 | 22 | 6 |  |
| >32 | 81(49.09) | 5(22.73) | 1(16.67) | >26.48 | | 82(49.7) | 6(27.27) | 1(16.67) |  |
| ≤32 | 84(50.91) | 17(77.27) | 5(83.33) | ≤26.48 | | 83(50.3) | 16(72.73) | 5(83.33) |  |
| **25% percentile of CtV as cutoffs** | | | | | | | | | |
| **hrHPV** | 446 | 76 | 34 | **hrHPV** | | 446 | 76 | 34 |  |
| >26.2 | 332(74.44) | 45(59.21) | 17(50) | >18.06 | | 334(74.89) | 35(46.05) | 7(20.59) |  |
| ≤26.2 | 114(25.56) | 31(40.79) | 17(50) | ≤18.06 | | 112(25.11) | 41(53.95) | 27(79.41) |  |
| **HPV16** | 99 | 41 | 25 | **HPV16** | | 99 | 41 | 25 |  |
| >25.7 | 74(74.75) | 25(60.98) | 13(52) | >14.77 | | 74(74.75) | 26(63.41) | 14(56) |  |
| ≤25.7 | 25(25.25) | 16(39.02) | 12(48) | ≤14.77 | | 25(25.25) | 15(36.59) | 11(44) |  |
| **HPV18** | 31 | 5 | 1 | **HPV18** | | 31 | 5 | 1 |  |
| >27.3 | 23(74.19) | 4(80) | 0(0) | >16.6 | | 23(74.19) | 4(80) | 0(0) |  |
| ≤27.3 | 8(25.81) | 1(20) | 1(100) | ≤16.6 | | 8(25.81) | 1(20) | 1(100) |  |
| **Non-16/18 hrHPV** | 316 | 30 | 8 | **Non-16/18 hrHPV** | | 316 | 30 | 8 |  |
| >26.1 | 236(74.68) | 17(56.67) | 4(50) | >20.51 | | 237(75) | 19(63.33) | 3(37.5) |  |
| ≤26.1 | 80(25.32) | 13(43.33) | 4(50) | ≤20.51 | | 79(25) | 11(36.67) | 5(62.5) |  |
| **A9** | 165 | 22 | 6 | **A9** | | 165 | 22 | 6 |  |
| >27.5 | 121(73.33) | 9(40.91) | 1(16.67) | >21.19 | | 123(74.55) | 11(50) | 1(16.67) |  |
| ≤27.5 | 44(26.67) | 13(59.09) | 5(83.33) | ≤21.19 | | 42(25.45) | 11(50) | 5(83.33) |  |
